# *Sporothrix brasiliensis*: Epidemiology, Therapy, and Recent Developments

**DOI:** 10.3390/jof9090921

**Published:** 2023-09-13

**Authors:** Melissa Orzechowski Xavier, Vanice Rodrigues Poester, Mariana Rodrigues Trápaga, David A. Stevens

**Affiliations:** 1 Programa de Pós-Graduação em Ciências da Saúde, Faculdade de Medicina (FAMED), Universidade Federal do Rio Grande (FURG), Rio Grande 96200-190, RS, Brazil; melissaxavierfurg@gmail.com (M.O.X.); vanicerp@gmail.com (V.R.P.); marianartrapaga@gmail.com (M.R.T.); 2 Laboratório de Micologia, Faculdade de Medicina (FAMED), Universidade Federal do Rio Grande (FURG), Rio Grande 96200-190, RS, Brazil; 3 California Institute for Medical Research, San Jose, CA 95128, USA; 4 Division of Infectious Diseases and Geographic Medicine, Stanford University Medical School, Stanford, CA 94305, USA

**Keywords:** sporotrichosis, antifungal, zoonosis, cats, subcutaneous mycosis, drugs

## Abstract

Sporotrichosis caused by *Sporothrix brasiliensis* is an emergent mycosis that is now a worldwide concern. One important step to sporotrichosis control is its correct treatment. However, limitations abound; thus, new antifungals, mainly focused on *S. brasiliensis*, are urgently needed. We performed a systematic review (following the PRISMA guideline) focused on (1) the global distribution of human and animal sporotrichosis by *S. brasiliensis*, especially outside of Brazil; (2) appraising therapies tested against this pathogen. We identified sporotrichosis caused by *S. brasiliensis* reported in five countries (Paraguay, Chile, Argentina, the United Kingdom, and the United States) in addition to Brazil, occurring on three continents, highlighting the epidemiological scenario in Argentina with an important increase in reported cases in recent years. Regarding the antifungal activity of drugs, 25 articles described the in vitro action of 20 unique chemicals and eight repurposed drugs against *S. brasiliensis*. Only five studies reported in vivo activity against *S. brasiliensis* (five drugs) using invertebrate and vertebrate models. Sporotrichosis caused by *S. brasiliensis* has a global impact and it is no longer specifically a Brazilian problem. We review the need for understanding the disease epidemiology, education of clinicians and of the populace, organization of health care delivery to respond to a spreading epidemic, and research on therapy for sporotrichosis.

## 1. Introduction

Sporotrichosis, caused by the dimorphic fungi *Sporothrix* spp., is the main subcutaneous mycosis with a worldwide distribution. For many years, sporotrichosis was associated with only one species, *S. schenckii*, and was linked to traumatic injury in people who manage soil, plants, and flowers; it was even known as “Gardener Disease” due to the importance of the environment as a source of infection [1,2,3]. However, in the 1990s in Brazil, especially in the Rio de Janeiro (RJ) and Rio Grande do Sul (RS) states, a different epidemiological pattern of sporotrichosis began to occur, and an increase in cases transmitted by sick cats through scratches or bites was noted [4,5,6,7,8,9]. 

Since then, outbreaks of sporotrichosis transmitted by cats have been described throughout the entirety of Brazil, with the worst epidemiological scenario occurring in RJ, and in second place RS, where thousands of cases were described in recent decades [9,10,11]. In contrast to the scenario of sporotrichosis acquired from the environment, in which sporadic and dispersed cases are predominant, sporotrichosis transmitted by cats is quickly dispersed through animal and human communities in a short period of time, indicating a severe public health problem [10,12]. Cats are prominent victims of infection by *S. brasiliensis*, and develop a severe presentation of sporotrichosis with copious fungal cells in their lesions. This high fungal load, in addition to the social behavior of cats, which frequently fight, allows cats to easily transmit the fungus to other individuals, disseminating the disease inter- and intraspecies. Mutual grooming of cats may also play a role in intraspecies transmission [2,13]. 

Dogs and other animals (domestic and wild) can develop sporotrichosis, however in these animals, the disease follows the pattern described in humans: a predominance of milder cutaneous cases [8,13]. It is speculated that canine cases may arise from disputes with cats. An intriguing question concerns the rates of *S. brasiliensis* infection in various inbred strains of domestic cats and in wild felines, and data are needed in the scientific literature. In addition, identification of the genes that control resistance to infection in cats, humans, and experimental animals is of interest.

Concurrent with the emergence of zoonotic sporotrichosis in Brazil, advances in studies allowed for the correct identification of *Sporothrix* isolates via phenotypic characterization and molecular targets [1,14]. It was shown that *Sporothrix schenckii* actually was a complex of species, morphologically similar, but genotypically distinct [1,14]. Today, it is understood that species of the *Sporothrix* complex associated with clinical cases are *S. schenckii*, *S. brasiliensis*, *S. globosa*, *S. luriei*, *S. mexicana*, *S. pallida*, and *S. chilensis*. The first three are the main etiological agents of sporotrichosis worldwide, identified in more than 90% of the cases reported [1,3,14,15,16].

Presently, it is also established that *S. brasiliensis* is the species responsible for the hyperendemicity of cat-transmitted sporotrichosis in Brazil, while *S. schenckii* and *S. globosa* are the species mostly associated with an environmental infection [2,13,16,17,18]. *S. schenckii* and *S. globosa* are widely dispersed, occurring on all continents, with *S. globosa* more dominant on the Asian continent [16,17,18]. On the other hand, *S. brasiliensis*, until 2018, was supposedly restricted to Brazil; however, in recent years, this species has been identified in autochthonous cases from other Latin American countries (Argentina, Paraguay, Chile) as well as in Europe and in the United States of America, via imported cats from Brazil [10,19,20,21,22,23,24,25]. Thus, it is essential to understand the reach of *S. brasiliensis* in the world, given that when it is introduced into a cat population, this fungus can quickly be dispersed, causing a severe public health problem [12].

Sporotrichosis is defined as an implantation mycosis, predominantly manifesting as cutaneous or lymphocutaneous sites in human patients [5,26]. In the hyperendemic scenario caused by *S. brasiliensis*, a new pattern in humans of more severe cutaneous lesions is reported, as well as extracutaneous manifestations such as ocular, nasal, pulmonary, and meningeal diseases, and/or hypersensitivity manifestations. These can be seen in immunocompetent patients, but the disseminated presentation is more frequent in immunocompromised persons [26,27,28,29,30,31,32,33,34]. Indeed, more severe disease can be reflected in an increase in the rate of human hospitalizations due to sporotrichosis, as shown in the RJ hyperendemic area through the years associated with the increase in zoonotic cases [35]. With respect to the more severe sporotrichosis infections seen in immunocompromised humans, it is also of interest whether cats with leukemia also have more severe disease. Co-infection of retroviruses associated with immunodeficiency in cats, Feline Immunodeficiency Virus (FIV) and Feline Leukemia Virus (FeLV), with *S. brasiliensis* was evaluated in a few studies, demonstrating a likely association of the co-infection with more severe clinical manifestations. However, this association is not yet proven [36,37]. Leukemia in cats is not an uncommon disease; for example, in the USA, up to 3% may have some stage of leukemia [38].

A significant step to control feline and zoonotic sporotrichosis is the correct treatment of infected cats. However, a low rate of cure of feline sporotrichosis with the drug of choice (itraconazole—ITZ) (40% to 77%) is reported, and similarly, the combination of ITZ with other antifungals (potassium iodide or amphotericin B) is also not a beneficial strategy to achieve therapeutic success (48% and 73%, respectively) [36,39,40,41,42]. In addition, high costs and a high rate of side effects associated with the necessity of a long period of treatment, requiring oral drug administration once a day, result in a high rate of treatment interruption by cat owners and, even worse, ends with the abandonment of the animals on the streets [36,39,40,41,42].

ITZ is also the drug of choice for human sporotrichosis, providing a satisfactory rate of cure (around 90%); however, side effects are also frequently reported [43,44]. In addition, in the hyperendemic of sporotrichosis caused by *S. brasiliensis* in RS, the necessity to increase the doses of ITZ above the 200 mg/day previously described in the international guidelines [45] to 400 mg/day to obtain clinical cure was reported [44]. 

Alternative options for sporotrichosis treatment are restricted to amphotericin B, potassium iodide, and terbinafine, according to international guidelines [13,45]. Amphotericin B is nephrotoxic and an intravenous drug, which may necessitate hospitalization; potassium iodide is associated with frequent side effects, and terbinafine is still being evaluated in sporotrichosis cases [13,44,45,46,47]. Thus, new options for sporotrichosis treatment, mainly focusing on *S. brasiliensis*, are urgent. Various in vitro and in vivo studies have described new potential drugs against this *Sporothrix* species [48,49,50]. Chemical drugs are a promising area for research, with data regarding stability and other pharmacological characteristics of the molecules [48,49,50,51,52,53]. Repurposing drugs are a great strategy to search for new potential antifungals since their pharmacological characteristics are already known, and they ordinarily have demonstrated activity in in vitro tests against *S. brasiliensis* [51,52]. 

Therefore, considering the emergence of *S. brasiliensis* in Brazil and other Latin American countries, and its potential to spread to other continents, the severe impact that it can cause on public health indicates that this pathogen needs to be viewed as a global concern. The spread beyond its prior geographic restriction, showing the dissemination of *S. brasiliensis* to other countries, and the lack of health vigilance regarding this pathogen is worrying. Thus, this systematic review has two objectives: (1) show the distribution of human and animal sporotrichosis by *S. brasiliensis* described around the world, especially outside of Brazil; (2) review chemical therapies already tested (in vitro and in vivo) against this pathogen. 

## 2. Materials and Methods

We performed a systematic review, following the PRISMA guideline [54], and using the databases Pubmed, SciELO, Web of Science, and LILACS. To increase our search, references of selected articles were read to find potential articles to be included. Two groups of descriptors were used, one to find epidemiological cases caused by *S. brasiliensis* outside Brazil (objective 1), [*Sporothrix*] and [*brasiliensis*] OR [*Sporothrix*] and [zoonosis] OR [*Sporothrix*] and [cat] OR [*Sporothrix brasiliensis*] and [epidemiological], and another to evaluate new potential therapies against *S. brasiliensis* (objective 2), [*Sporothrix brasiliensis*] and [in vitro] OR [*Sporothrix brasiliensis*] and [microdilution] OR [*Sporothrix brasiliensis*] and [macro-dilution] OR [*Sporothrix brasiliensis*] and [repurposing drug] OR [*Sporothrix brasiliensis*] and [chemical] OR [*Sporothrix brasiliensis*] and [in vivo] OR [*Sporothrix brasiliensis*] and [animal model] OR [*Sporothrix brasiliensis*] and [antifungal]. 

In this study we included articles (1) published in English, Portuguese, or Spanish and (2) published during the period of 1990 to May 2023. Articles were selected by the title, abstract, and/or by a full reading of the text, to cover both objectives. For objective 1, we included articles with (3) human or animal sporotrichosis cases from outside Brazil, with *S. brasiliensis* identified via molecular techniques, or (4) human or animal sporotrichosis cases from outside Brazil which inferred cat-transmission of *S. brasiliensis* (cats imported from Brazil). Data obtained from outside cases were grouped with an estimated number of human and animal sporotrichosis cases in Brazil based on a recent systematic review, which described the historical development of sporotrichosis in the country between 1990 and 2020 [3]. Manifestation of cutaneous sporotrichosis injuries in human patients were classified as fixed (only one lesion without lymph node impairment) or lymphocutaneous (two or more lesions with lymph node impairment) [26]. For objective 2, articles included were those that (5) described results of in vitro or in vivo experiments with any chemical drug against *S. brasiliensis* and were restricted to in vitro tests that used international protocols (Clinical and Laboratory Standards Institute—CLSI or European Committee on antimicrobial susceptibility testing–EUCAST); review articles and studies with natural extracts were excluded. 

Data of the two different objectives were added to Excel software (Microsoft Corporation^®^, Redmond, WA, USA) in two different files to analyze. For objective 1, the number of human and feline sporotrichosis cases diagnosed outside of Brazil and an estimate of the number of cases that occurred in Brazil were considered with the geographical coordinates obtained from Google maps (https://maps.google.com/, accessed on 17 July 2023). QGIS software (Open Source Geospatial Foundation—OSGeo) was used to analyze the geographical distribution of cases; for this, the geographic reference was superimposed on a world map, generating a Geographic Information System (GIS). For objective 2, the results of minimal inhibitory concentration (MIC) values were compiled for each individual drug.

## 3. Results

### 3.1. Sporotrichosis Caused by S. brasiliensis Outside of Brazil

**Figure 1:**
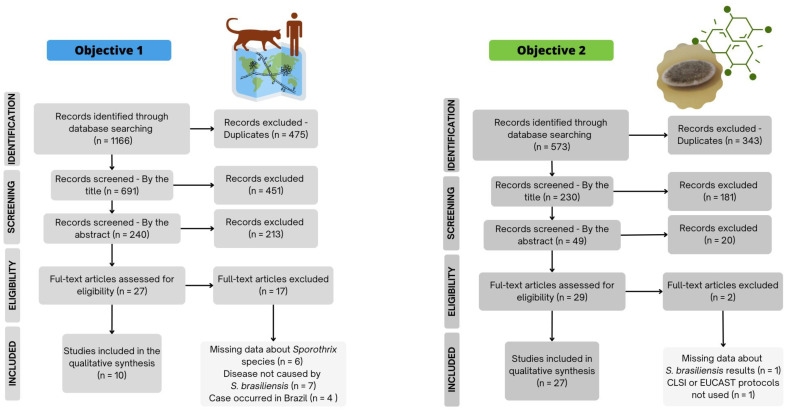
Flowchart describing the total of scientific articles obtained by the search, and those included with regard to the two objectives of our study: (1) evaluate the global distribution of sporotrichosis by *Sporothrix brasiliensis*; (2) evaluate potential new chemical therapies studied in vitro and in vivo against this pathogen. CLSI: Clinical and Laboratory Standards Institute; EUCAST: European Committee on antimicrobial susceptibility testing.

**Figure 2:**
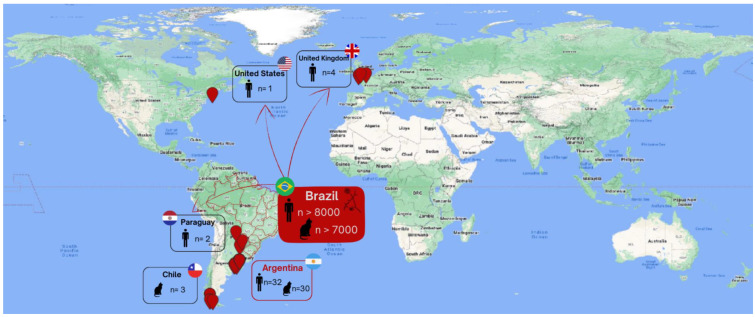
A world map showing sporotrichosis caused by *Sporothrix brasiliensis*. Each human icon represents human cases, and cat icon, feline sporotrichosis cases. n: number.

The bibliographic search for objective 1 returned 1166 articles, with 475 duplicated, and the majority eliminated by the title or by the abstract. Twenty-six resulted in a review of the full text, resulting in including ten articles (Figure 1). In this cohort, we identified sporotrichosis caused by *S. brasiliensis* reported in five countries outside of Brazil, occurring on three continents: South America (Paraguay, Chile, and Argentina), Europe (the United Kingdom), and North America (the United States) (Figure 2) [19,20,21,22,23,24,25,55,56,57].

Cases reported in Chile, Argentina, and the United Kingdom (UK) had the etiological agent identified via molecular methods as *S. brasiliensis*, and were considered as proven cases [20,21,23,24,25,55,56,57]. On the other hand, molecular identification of the etiological agent to species level in cases from the United States (USA) and Paraguay [19,22] was not described, but these cases were considered as probable *S. brasiliensis* sporotrichosis, taking into consideration that all of them (n = 3) were transmitted by infected cats imported from Brazil.

In addition to Brazil, where sporotrichosis caused by *S. brasiliensis* numbers thousands of cases and occurs in practically all regions of the country [3], Argentina in the last decade showed a marked increase in the number of confirmed cases of this zoonotic mycosis, comprising 50% (n = 5) of the papers included in this review. Until 2011, sporadic cases of sporotrichosis were reported in Argentinian territory; however, in the last decade a worrisome increase in this diagnosis was described, with a total of more than 60 cases (n = 30 in cats and n = 32 in humans) identified throughout the country, already covering five provinces (Misiones, Buenos Aires, Chaco, El Calafate, Santa Cruz) [20,21,23,55,57]. Moreover, a retrospective study with molecular analyses of *Sporothrix* spp. isolated from human sporotrichosis cases dated from the 1980s from this country proved that *S. brasiliensis* was already present in Argentina in that decade. Notably, these isolates came from cases occurring in a border region of Brazil, the Misiones province. In the same study, *S. brasiliensis* was also identified in isolates recovered from the soil of the Chaco Province [20]. Sporotrichosis case reports from Argentina only describe cutaneous or lymphocutaneous presentation of this mycosis.

From Paraguay, one study described two human cases of sporotrichosis transmitted by an infected cat transported from Brazil in 2017. Both cases occurred in the same family (father and son). They developed a fixed or lymphocutaneous manifestation of this mycosis [19]. In Chile, the first paper describing feline sporotrichosis cases caused by *S. brasiliensis* was published in 2023, reporting three cases of infected cats (two domestic and one feral cat) in the Magallanes region [25]. Human cases have not been reported there as yet.

Outside of South America, in 2020 the first USA zoonotic sporotrichosis case probably caused by *S. brasiliensis* (not identified at the species level) was reported in a female patient who returned from Brazil. During her time in Brazil she was bitten by a cat, developing cutaneous lesions in the infected hand. Samples were collected and resulted in a culture of *Sporothrix* spp. [22]. More recently, UK zoonotic sporotrichosis cases proven to be caused by *S. brasiliensis* (identified via molecular methods) were reported in two articles describing fungus transmission; in these reports, one veterinarian and two family members (owner and owner’s daughter), who developed the fixed or lymphocutaneous forms of the disease, were infected by a cat imported from Brazil [24,56].

### 3.2. Drugs Tested (In Vitro and In Vivo) against This Pathogen

The search for articles to be included in objective 2 (therapies against *S. brasiliensis*) initially returned 573 articles. The flowchart (Figure 1) shows that after exclusion criteria (343 were duplicates, and 201 eliminated by reading the title or the abstract), 27 were read in full and included. 

#### 3.2.1. In Vitro Studies

Regarding in vitro activity, 25 articles described the inhibitory activity of 28 drugs against *S. brasiliensis* (100% fungal-inhibition criterion): 20 chemicals and 8 repurposed drugs [48,49,51,53,58,59,60,61,62,63,64,65,66,67,68,69,70,71,72,73,74,75,76,77,78]. Data about the MIC for each individual drug are shown in Table 1. In addition to the inhibitory fungal activity, six papers showed that pentamidine, miltefosine, chitosan, naphthoquinones derivative, and olorofim also inhibited biofilm formation of *S. brasiliensis* [58,60,63,64,74,78].

#### 3.2.2. Combination Therapy

**Figure 3:**
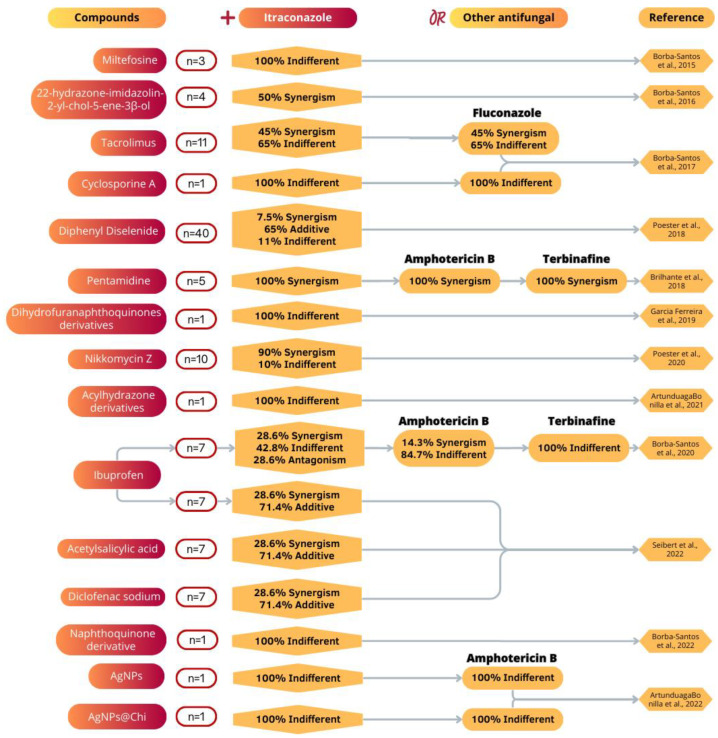
Combination of drugs against *Sporothrix brasiliensis* described in the scientific literature. N: number of isolates. AgNPs: silver nanoparticles; AgNPs@Chi: silver nanoparticles with chitosan. Reference: Borba-Santos et al., 2015 [62]; Borba-Santos et al., 2016 [66]; Borba-Santos et al., 2017 [51]; Poester et al., 2018 [48]; Brilhante et al., 2018 [58]; Garcia Ferreira et al., 2019 [69]; Borba-Santos et al., 2020 [49]; Poester et al., 2020 [53]; Artunduaga Bonilla et al., 2021 [71]; Artunduaga Bonilla et al., 2022 [70]; Borba-Santos et al., 2022 [63]; Seibert et al., 2022 [75].

In addition, 12 articles evaluated the activity of 14 drugs together with commercial antifungals used for the treatment of cats or humans (ITZ, terbinafine, amphotericin, and fluconazole). Different interaction effects occurred (synergism, additive, indifferent, antagonism), ranging from 14.3% to 100% beneficial effects, which are described in Figure 3 [48,51,53,58,62,63,65,66,69,70,71,75].

#### 3.2.3. In Vivo Studies

**Figure 4:**
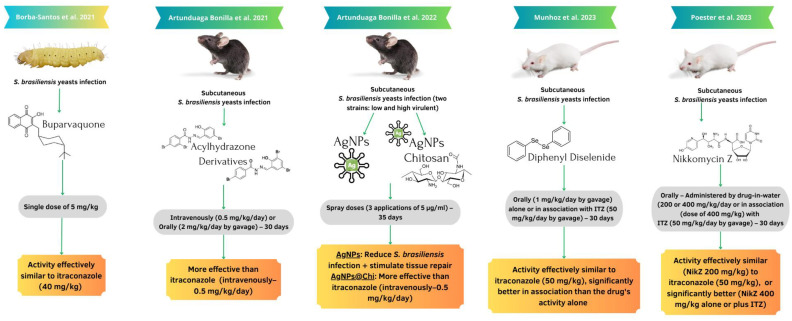
In vivo studies regarding anti-*Sporothrix brasiliensis* activity described in the scientific literature. AgNPs: silver nanoparticles; Chi: chitosan. Reference: Borba-Santos et al., 2021 [61]; Artunduaga Bonilla et al., 2021 [71]; Artunduaga Bonilla et al., 2022 [70]; Munhoz et al., 2023 [50]; Poester et al., 2023 [79].

Only five studies described in vivo tests. Five chemical entities were studied, including buparvaquone (a repurposed drug), silver nanoparticles (AgNPs) with and without chitosan (Chi), two acylhydrazone derivatives (D13 and SB-AF-1002), diphenyl diselenide, and nikkomycin Z [50,61,70,71,79] (Figure 4), in the treatment of experimental sporotrichosis by *S. brasiliensis*. One drug was tested in an invertebrate model, *Galleria mellonella* (buparvaquone drug), and four in a murine model, two using C57BL/6 mice and two BALB/cJ. 

The only study using an alternative model (invertebrate animal) of sporotrichosis by *S. brasiliensis* was conducted with *G. mellonella*, which were infected and then treated with buparvaquone by a single dose of 5 mg/kg or 10 mg/kg. At 5 mg/kg, buparvaquone showed similar activity to ITZ regarding fungal burden reduction, and it resulted in a lower mortality rate [61]. The other four studies describing potential drugs with in vivo activity against sporotrichosis by *S. brasiliensis* were developed in vertebrate models via a subcutaneous infection of *S. brasiliensis* yeast cells, followed by 30–35 days of treatment [50,70,71,79]. 

Acylhydrazone derivatives administered intravenously (0.5 mg/kg/day, via tail) or orally (2 mg/kg/day by gavage) showed more effective activity in either route than the ITZ treatment (intravenously, 0.5 mg/kg/day); the treatment with one of the derivatives (D13 drug) resulted in less mortality in animals compared with all other groups [71]. AgNPs with and without Chi were administered via spray applications onto sporotrichosis lesions (three times/day of 5 μg/mL of silver) using experimental models of a low or high virulence of *S. brasiliensis*. In both models, treatment with AgNPs, or especially with AgNPs + Chi, resulted in paw-swelling reduction, probably due to decreased inflammatory response, and the prevented mortality of animals in comparison with the ITZ group (intravenously, 0.5 mg/kg/day) [70]. Accordingly, diphenyl diselenide orally administered in a dosage of 1 mg/kg had a similar effect to the ITZ treatment (50 mg/kg/day by gavage) regarding fungal burden in tissues, and its use together with ITZ showed significantly better results than the use of either drug alone [50]. Nikkomycin Z (NikZ) was also indicated as a potential new antifungal drug against *S. brasiliensis*. NikZ, administered in the drinking water of the animals at a dose of 400 mg/kg/day, produced better results than ITZ (50 mg/kg/day by gavage). The use of both drugs together was better than each alone in reducing the fungal burden in infected tissues [79].

## 4. Discussion

Since the beginning of the emergence of sporotrichosis caused by *S. brasiliensis* three decades ago, the transmission scenario in Brazil has expanded, and it is presently critical and out of control. This country has, presently, more than fifteen thousand cases reported in the literature, which is much lower than the actual total number of cases, since the disease is not included in the national list of diseases requiring compulsory notification [3]. Recently (22 May 2023), a technical note was published from the Health Ministry of Brazil, not yet including this mycosis in the list of notifiable diseases, but stipulating measures of control and surveillance to all of the country [80]. 

Expanding on the Brazilian situation, the disease is now reported in bordering countries of Brazil, Argentina and Paraguay, and on three continents, reinforcing that public health measures must be implemented [19,20,21,22,23,24,25,55,56,57]. Moreover, *S. brasiliensis* infection has already spread to another bordering country (Uruguay) of Brazil (personal communication), causing proven cases in humans and cats. Thus, it is likely this species also spread to other neighboring countries, which have not reported cases yet, such as Peru, Colombia, and Venezuela. 

In the Brazilian hyperendemic, it was suggested that *S. brasiliensis* evolved from independent geographical sources: the main epicenters of the disease occur in both the southeast and southern regions [81,82,83]. Regarding cases outside these areas, it is vital to evaluate the molecular characteristics of strains to determine if *S. brasiliensis* also has had a genetic evolution in these countries with adaptation to cats, or if it was exported from Brazil with a clonal distribution. In Argentina, *S. brasiliensis* was detected early in our awareness of this pathogen, with cases retrospectively dating from the 1980s (including environmental detection of the pathogen), possibly suggesting that in this country this species had an evolution independent from that in Brazil [20].

However, even if individual genotypic evolution among countries occurred, *S. brasiliensis* was also proven to be exported (cases in Paraguay, the UK, and the USA) or probably exported (Argentina and Chile) to other countries from Brazil. The spreading of *S. brasiliensis* to bordering Brazilian countries probably occurred due to the free passage of cats (domestic and wandering) across borders in these regions [19,20,21,22,23,24,25,55,56,57]. Extreme southern regions from Argentina and Chile (Patagonian and Magallanes regions, respectively), where sporotrichosis cases were detected in recent years, could indicate a recent adaptation of the pathogen or could be associated with the transportation of *S. brasiliensis* in cargo ships that travel to different countries and often carry cats to control the infestation of rodents, contributing to the transport of infected cats among international sites [23,25]. 

Therefore, sporotrichosis should be noted in animal transit travel guides, traveling animals inspected for disease, and security in marine travel improved, all these regarding the transport of cats, particularly in Brazilian routes. This is especially important in Brazilian connections in the areas of the country hyperendemic for *S. brasiliensis*. Another hypothesis, which should be experimentally tested, regards the possible role of feline parasites (fleas, worms) in *S. brasiliensis* transmission as a vector from infected animals to other animals, and even humans.

Regarding clinical manifestations of zoonotic sporotrichosis outside of Brazil, the majority of human cases were classical cutaneous (fixed or lymphocutaneous) cases of the disease, which helps in early suspicion and diagnosis, as well as in the favorable outcome of patients [19,20,21,22,23,24,25,55,56,57]. However, the progression in the epidemiological pattern of this mycosis, as occurred in Brazil, also brought an increase in atypical and more severe human manifestations of the disease (ocular, nasal, pulmonary, meningeal, and/or hypersensitivity reactions), which have a more unfavorable prognosis; this emphasizes the need for highly effective therapy [26,27,28,30,31,32,33,34].

To this end, with regard to new promising drugs with anti-*S. brasiliensis* activity, approximately 30 drugs with in vitro inhibitory activity have been described. In addition, some drugs showed anti-biofilm activity of *Sporothrix* spp., and/or potentiated the activity of commercial antifungal drugs (mainly ITZ, the drug of choice for sporotrichosis treatment), stressing the need for continuing studies in this field of new drug development [48,49,51,53,58,59,60,61,62,63,64,65,66,67,68,69,70,71,72,73,74,75,76,77,78]. 

Only five in vivo studies of new potential treatments for sporotrichosis by *S. brasiliensis* were found in the literature [50,61,70,71,79]. Buparvaquone was the only drug tested in an invertebrate model, a naphthoquinone class drug used as an antiprotozoal therapy, with a mechanism of action suggested as interrupting the respiratory process of parasites [61,84,85]. We suggest that laboratory studies of therapy in cats (in contrast to studies in felines in the field) offer the opportunity for controlled circumstances to investigate the efficacy of promising drugs and their pharmacology. 

In vertebrate models (mice), four classes of drugs were tested, two acylhydrazone derivatives (D13 and SB-AF-1002) and two types of AgNPs (pure or with Chi-AgNPs@Chi), diphenyl diselenide and NikZ [50,70,71,79]. Acylhydrazone drugs act in the vesicular transport and the cell cycle of fungi, indirectly impacting glucosylceramide (GlcCer) synthesis. The derivatives tested for the treatment of experimental sporotrichosis (D13 and SB-AF-1002) were shown in previous studies to have low toxicity for mammalian cells [85,86]. Silver and chitosan molecules show tissue-repair properties, reported in other studies, and are potential anti-inflammatory drugs; the activity of both on nanoparticle presentation against *S. brasiliensis* reaffirmed this effect [87,88]. The antifungal activity of AgNPs occurred on the fungal cell wall, influencing the strength and permeability of this structure, and introduced the potential of using silver as a spray and as a nanoparticle to reduce the toxicity of this element [89,90,91].

Diphenyl diselenide is an organoselenium drug with many previous pharmacological studies. It has a low toxicity with daily oral doses (30 μg) in long periods of administration (8 months) in rabbits, proven in an animal model [92]. It is proposed that its activity is associated with the antioxidant mechanism of fungal defenses, due to the fact that this molecule imitates the mechanism of glutathione peroxidase [93]. NikZ is a nucleoside-peptide that acts as a competitive inhibitor of chitin synthase; chitin is a fungal cell-wall component [94]. The drug showed low toxicity in high doses in a mouse model (1000 mg/kg twice daily) [95], confirmed in human studies thus far (up to 2000 mg/day) [96]. 

We note that diphenyl diselenide and NikZ, in addition to their in vivo activity alone against *S. brasiliensis*, showed a synergistic effect with ITZ therapy [50,79]. NikZ has the advantage that it can be administered in the drinking water of animals, which can be a very promising application for sporotrichosis therapy, considering that one of the obstacles to therapy in cats is the use of an oral drug every day for several months [13,79]. Thus, future field studies should investigate the NikZ activity against *S. brasiliensis* in naturally infected cats in hyperendemic areas.

In view of the possible high impact of *S. brasiliensis* globally, we investigated the measures made in Brazil, the epicenter of the disease, to control the dissemination of this species, in approximately three decades of hyperendemicity. We took as our departure the proposal of measures by Barros et al. [97], which was the first publication to summarize this public health problem, and suggest possible strategies for control. Briefly, five facets of the problem were detected: (1) lack of a public health program to control sporotrichosis; (2) lack of an available sporotrichosis drug (at low cost or without cost) for animal and human sporotrichosis; (3) lack of publicly accessible places to diagnose and treat animal sporotrichosis cases; (4) population unfamiliarity about sporotrichosis; (5) multifactorial difficulty in treating infected cats. 

**Figure 5:**
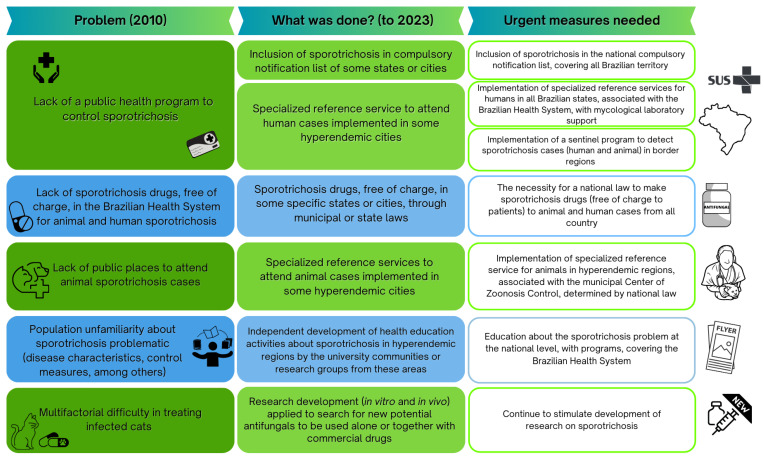
Problems generated by hyperendemicity of sporotrichosis caused by *Sporothrix brasiliensis* in Brazil and proposed actions to face this reality throughout three decades of this epidemiological situation, problematic based on the description by Barros et al., 2010 [97].

Some measures have been taken in facing these problems, as described in Figure 5. It was possible to identify individual efforts in hyperendemic regions. In 2010, the lack of public health programs to control sporotrichosis was noted; presently, several cities or states included sporotrichosis as a disease requiring compulsory notification at the municipal or state level, such as Minas Gerais, Salvador, Rio de Janeiro, São Paulo, Distrito Federal, and Paraná [98,99,100,101,102]. Another strategy is the implementation of a specialized reference service to diagnose suspected sporotrichosis cases, such as the service reported in RJ, the first state to implement a human and animal service to centralize attention to cases, and the service established by our research group in a city in the southern RS state [13,44,103,104]. However, an important goal to control sporotrichosis cases is the inclusion of the disease in the national compulsory notification list, allowing for knowledge of the true number of cases; the implementation of public health measures at the national level, emphasizing the availability of a drug (free-of-charge) for sporotrichosis treatment for humans and cats (until now only available in certain regions); and the implementation of a service to attend to infected animals throughout the country, especially in hyperendemic areas, such as the service described by Moreira et al. [105] in a municipality of Minas Gerais state, Brazil.

One of the problems associated with sporotrichosis control already highlighted in 2010 is the unfamiliarity of the population and health professionals with the disease, including basic knowledge about its existence [106,107]. Now we can indicate the independent development of health education activities about sporotrichosis in hyperendemic regions by the university community and research groups from these areas or by local public entities, producing educational material and creating education activities for the local population [104,106,107,108]. Another concern is the effective treatment of infected animals in view of limitations to the use of the drug of choice. We urge the continued development of research for new potential drugs with anti-*S. brasiliensis* activity, the second objective of this report. 

## 5. Conclusions

In conclusion, our systematic review summarizes the global impact of sporotrichosis caused by *S. brasiliensis*; it is no longer a Brazilian problem, but, presently, a world concern. Urgent measures to control the dissemination of this disease in the national and international territories of bordering countries are necessary. There must be investments in public health surveillance, mainly at the national level, with the inclusion of sporotrichosis as a disease requiring compulsory notification. At the international level, educational and research activities must develop to increase and spread knowledge about this important zoonosis. In addition, we compiled the available information regarding the search for potential antifungal drugs against *S. brasiliensis*; that research could, in the future, contribute to the epidemiological control of the disease.

## Figures and Tables

**Table 1:** Drugs and their in vitro antifungal activity against *Sporothrix brasiliensis* isolates.

Drug	Range of Concentrations	N *	Range MIC	Reference
22-hydrazone-imidazolin-2-yl-chol-5-ene-3β-ol	0.06–4 µg/mL	16	0.03–0.5 µg/mL	[66]
Tacrolimus	0.008–16 µg/mL	1	1 µg/mL	[51]
Cyclosporine A	0.125–16 µg/mL	1	1 µg/mL	
Diphenyl Diselenide	0.25 to 128 µg/mL	40	4–32 µg/mL	[48]
Pentamidine	0.03–16 µg/mL	10	0.13–1 µg/mL	[58]
Miltefosine	0.0312–16 µg/mL	48	0.5–2 µg/mL	[59]
	0.25–16 µg/mL	13	1–2 µg/mL	[62]
	0.0313–16 µg/mL	10	1–4 µg/mL	[60]
	0.03−16 µg/mL	3	0.5–4 µg/mL	[78]
Pyrazinoic acid	0.006–3.18 mg/mL	1	1.59 mg/mL	[67]
Pyrazinoic acid derivatives	0.005–5.1 mg/mL	5	0.05–3.06 mg/mL	
Dihydrofuranaphthoquinone derivatives (n = 9)	0.06–32 μM	1	4–32 μM	[69]
Nikkomycin Z	6.25–400 µg/mL	3	100–400 µg/mL	[53]
Acylhydrazone derivatives (n = 3)	0.06–4 µg/mL	1	0.25–2 µg/mL	[71]
Buparvaquone	0.005–2.61 µg/mL	20	0.005–0.16 µg/mL	[61]
Ibuprofen	2–1.024 µg/mL	7	128–512 µg/mL	[49]
	0.06–8 mg/mL	6	0.12–8 mg/mL	[75]
Naphthoquinone derivative (n = 5)	0.06–32 μM	1	2–32 μM	[63]
Acetylsalicylic acid	0.06–8 mg/mL	6	0.25–8 mg/mL	[75]
Diclofenac sodium	0.06–8 mg/mL	2	<0.06–2 mg/mL	
Complexes coordinated with cobalt (n = 3)	4–256 µg/mL	27	32–128 µg/mL	[76]
Hydrazone derivatives (n = 3)	Data not shown	7	2.7–13.3 µg/mL	[77]
Quinone derivatives (n = 11)			32–128 µg/mL	
Olorofilm	0.0002–1 μM	1	0.06 μM	[64]
Chitosan	2–512 µg/mL	10	16–128 µg/mL	[74]
Pentathiepin derivatives (n = 3)	Data not shown	8	0.5–8 µg/mL	[68]
Zinc ITZ complexes	0.04–204 μM	1	0.08–0.3 μM	[72]
Metal complexes with KTZ and CTZ	0.008–4 μM	1	0.25 μM	[73]
Diaminoquinazoline derivative	0.0002–1 μM	5	0.25–1 μM	[65]
Iodoquinol		5	0.5 μM	
Azole derivative		5	0.25–1 μM	
Silver nanoparticles Silver nanoparticles with chitosan	0.12–16 μg/mL	1	0.12 µg/mL	[70]
			0.5 µg/mL	

N: number of isolates; * including only the number of isolates for which an inhibitory activity of the drug was shown. MIC: minimal inhibitory concentration. ITZ: itraconazole; KTZ: Ketoconazole; CTZ: Clotrimazole.

## Data Availability

Not applicable.
